# Insect immunity in the Anthropocene

**DOI:** 10.1111/brv.13158

**Published:** 2024-11-05

**Authors:** Md Kawsar Khan, Jens Rolff

**Affiliations:** ^1^ Institute of Biology Freie Universität Berlin Königin‐Luise‐Str. 1‐3 Berlin 14195 Germany; ^2^ School of Natural Sciences Macquarie University 18 Wally's Walk, North Ryde‐2109 Sydney NSW Australia

**Keywords:** global change, host–pathogen interactions, insect decline, vector‐borne diseases, pollution

## Abstract

Anthropogenic activities result in global change, including climate change, landscape degradation and pollution, that can alter insect physiology and immune defences. These changes may have contributed to global insect decline and the dynamics of insect‐transmitted diseases. The ability of insects to mount immune responses upon infection is crucial for defence against pathogens and parasites. Suppressed immune defences reduce fitness by causing disease‐driven mortality and elevated immune responses reduce energy available to invest in other fitness traits such as reproduction. Understanding the impact of anthropogenic factors on insect–pathogen interactions is therefore key to determining the contribution of anthropogenic global change to pathogen‐driven global insect decline and the emergence and transmission of insect‐borne diseases. Here, we synthesise evidence of the impact of anthropogenic factors on insect immunity. We found evidence that anthropogenic factors, such as insecticides and heavy metals, directly impacting insect immune responses by inhibiting immune activation pathways. Alternatively, factors such as global warming, heatwaves, elevated CO_2_ and landscape degradation can indirectly reduce insect immune responses *via* reducing the energy available for immune function. We further review how anthropogenic factors impact pathogen clearance and contribute to an increase in vector‐borne diseases. We discuss the fitness cost of anthropogenic factors *via* pathogen‐driven mortality and reduced reproductive output and how this can contribute to species extinction. We found that most research has determined the impact of a single anthropogenic factor on insect immune responses or pathogen resistance. We recommend studying the combined impact of multiple stressors on immune response and pathogen resistance to understand better how anthropogenic factors affect insect immunity. We conclude by highlighting the importance of initiatives to mitigate the impact of anthropogenic factors on insect immunity, to reduce the spread of vector‐borne diseases, and to protect vulnerable ecosystems from emerging diseases.

## I. INTRODUCTION

Human activities have been the major driver of global change for the last two centuries (Ruddiman, 2013; Lewis & Maslin, 2015). Anthropogenic activities such as agricultural practices have transformed the landscape (Raven & Wagner, 2021). Greenhouse gas emission from fossil fuel burning, agriculture and livestock farming have changed the composition of the atmosphere and contributed to global warming (Skea *et al*., 2022). The average global temperature has been increasing since the Industrial Revolution and is predicted to increase by 2.2–3.5 °C by the end of the 21st century (Lee *et al*., 2023). Extreme climatic events such as drought, flood, heatwaves and fire are becoming more frequent and intense as a consequence of climate change (Ebi *et al*., 2021; Skea *et al*., 2022). Anthropogenic activities are thereby altering ecological interactions such as interactions between hosts and pathogens (herein, we use “pathogens” in the broadest sense, as including parasites) and evolutionary outcomes such as species extinction (Turvey & Crees, 2019; Román‐Palacios & Wiens, 2020; Raven & Wagner, 2021).

**Fig. 1:**
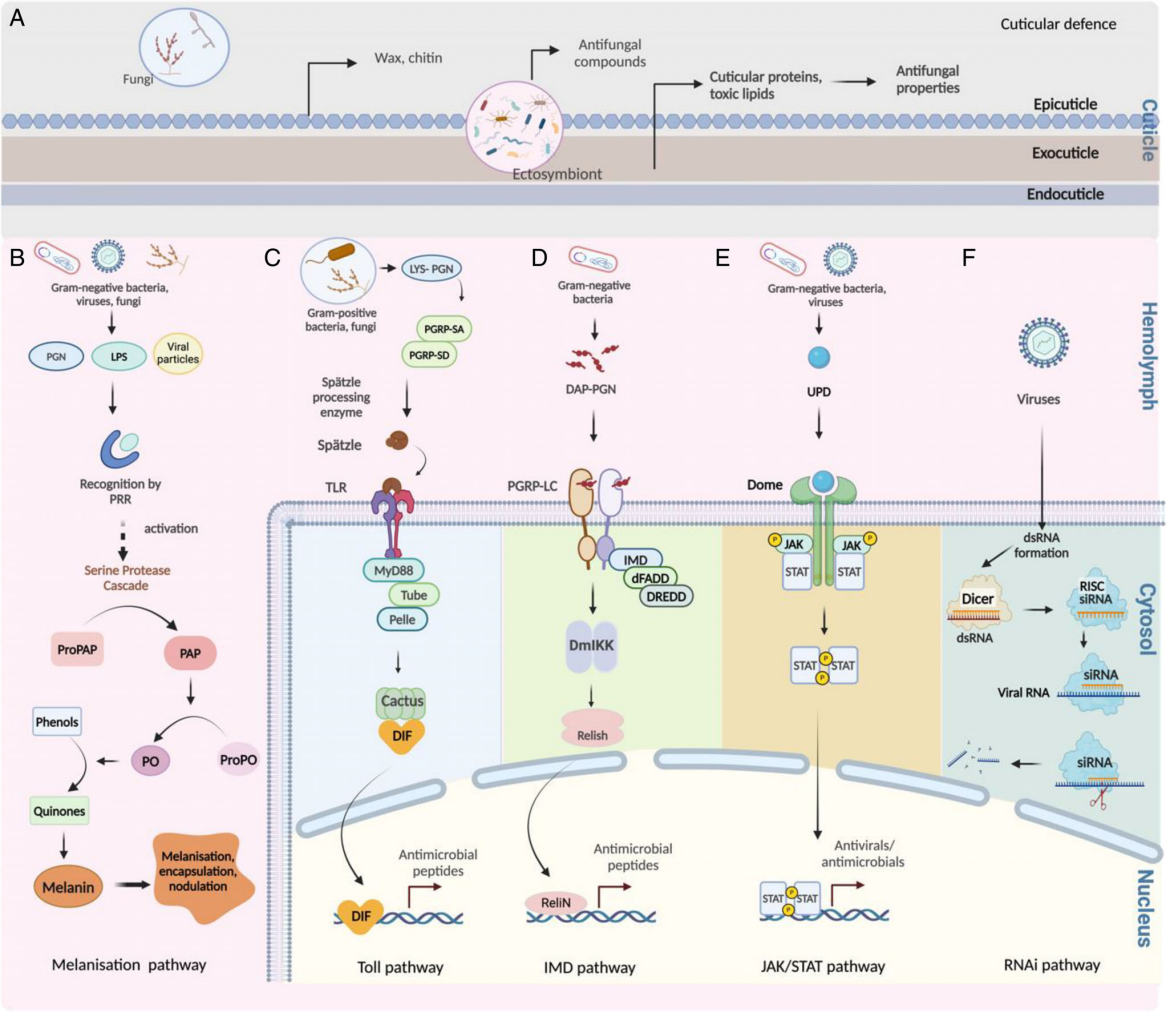
Insect immune pathways activated in response to parasites, fungi, bacteria and viruses. (A) The protective layer provided by the cuticle and associated microbiota. (B) The melanisation pathway. (C) The Toll pathway. (D) The IMD pathway. (E) The JAK–STAT pathway. (F) The RNAi pathway. While most of the components of these pathways were identified from studies on the fruit fly, insect immune pathways are highly conserved across species. DAP‐PGN, diaminopimelic acid‐containing peptidoglycan; dFADD, drosophila Fas‐associated death domain protein; DIF, forsal‐related immune factor; DmIKK, drosophila IκB kinase; DREDD, death‐related ced‐3/Nedd2‐like protein; dsRNA, double‐stranded RNA; IMD, immune deficiency; JAK/STAT, Janus kinase/signal transducers and activators of transcription; LPS, lipopolysaccharide; LYS‐PGN, lysine‐type peptidoglycan; MyD88, myeloid differentiation primary response protein 88; PAP, prophenoloxidase‐activating proteinase; PGN, peptidoglycan; PGR, peptidoglycan receptor; PO, phenoloxidase; PRR, pattern recognition receptor; ReliN, Relish N terminus; RISC, RNA‐induced silencing complex; RNAi, RNA interference; siRNA, small interfering RNA; TLR, Toll‐like receptor; UPD, unpaired. Figure created with BioRender.com.

Insects play essential roles in many ecosystems, including nutrient cycling, pollination, seed dispersal, and maintaining soil structure and fertility (Crespo‐Pérez *et al*., 2020). Insects are particularly vulnerable to human activity‐driven changes because of their short lifespan and sensitivity to environmental changes (Boggs, 2016; Halsch *et al*., 2021). Although there are variations among climatic zones and taxonomic groups, insect abundance is declining by approximately 1–2% every year (Dirzo *et al*., 2014; Hallmann *et al*., 2017; van Klink *et al*., 2020; Wagner *et al*., 2021). The decline of insect abundance in the Anthropocene is partly driven by direct mortality due to extreme climatic events, pollution and habitat loss (Wagner, 2020; Wagner *et al*., 2021). Anthropogenic activities, however, also impact insect immune responses (Fig. 1) and resistance to pathogens (St. Leger, 2021) which can increase pathogen‐driven mortality and may contribute to global species decline (Soroye, Newbold & Kerr, 2020; Harvey *et al*., 2023). The influences of Anthropocene‐specific environmental drivers on insect immune defence and pathogen resistance are likely complex and will depend on the specific taxon, local climate and the type and magnitude of anthropogenic disturbance. Identifying the key anthropogenic factors, and determining the extent of their impact, will be crucial to estimating disease‐driven extinction risk and to develop conservation strategies for protecting vulnerable insect species (Forister, Pelton & Black, 2019; Kawahara *et al*., 2021).

Understanding the impact of anthropogenic activities on insect defences against pathogens is not only important to protect insects from disease‐driven extinction risk, but also to determine potential changes in prevalence of vector‐borne diseases (Franklinos *et al*., 2019; Rocklöv & Dubrow, 2020). Insects are carriers of bacterial, parasitic and viral pathogens that cause widespread diseases in humans such as dengue, yellow fever, malaria, sleeping sickness, leishmaniasis, etc. (Franklinos *et al*., 2019). Stronger immune defences in insect vectors can limit the pathogen load and thereby reduce transmission to humans (Gabrieli *et al*., 2021). Anthropogenic activities could increase the prevalence of vector‐borne diseases by expanding the distribution range of vectors (Iwamura, Guzman‐Holst & Murray, 2020; Karypidou *et al*., 2020) but also by reducing insect immunity and resistance against pathogens. Recent studies have shed light on the impact of global change on insect immunity, yet our understanding of how global change‐driven alterations in insect immunity could impact transmission of vector‐borne diseases is rather limited.

In this review, we synthesise the impact of anthropogenic activities on insect immunity. First, we investigate studies where impacts of anthropogenic activities on insect immune responses and pathogen resistance have been tested experimentally. We then summarise the direction and magnitude of how different anthropogenic factors influence insect immune responses. We further synthesise how anthropogenic activities impact insect–pathogen interactions. Finally, we discuss the possible consequences of anthropogenic‐driven alterations in immunity and host–pathogen interactions on disease‐driven extinction risk in insects and on vector‐borne diseases in humans. Our review identifies that studies often focus on single anthropogenic factors instead of combinations and usually measure the impact of an anthropogenic factor on either insect immune response or pathogen resistance, but not both. We conclude by suggesting that future studies should determine the mechanism by which the anthropogenic factor impacts insect immunity, and by highlighting the importance of taking initiatives to mitigate anthropogenic factors to reduce the spread of vector‐borne diseases and to protect vulnerable ecosystems.

## II. BRIEF OVERVIEW OF THE INSECT IMMUNE SYSTEM

The insect immune system has three main components consisting of (*i*) physical barriers, (*ii*) cellular immunity and (*iii*) humoral immunity.

Physical barriers include the integument and cuticle layers in the gut. The integument, the outer surface of an insect, is formed by a single layer of cells (epidermis) covered by a multilayered cuticle (epicuticle, exocuticle and endocuticle) (Fig. 1A) (Coates *et al*., 2022). The cuticle is composed of chitin, wax and proteins and prevents invasion of environmental pathogens. The peritrophic membrane in the insect midgut and cuticle layers in the fore gut also contain chitin and protein, and function as a physical barrier against foodborne pathogens (Khan, Kojour & Han, 2023). Cuticle layers in insect integument and gut also harbour defensive symbionts that protect insects from pathogens (Fig. 1A) (Janke *et al*., 2022).

Cellular immunity is the first line of defence against pathogens after they enter the body. Cellular immune responses are carried out by hemocytes such as granular cells, crystal cells, oenocytoids, and plasmatocytes (Coates *et al*., 2022). Cellular immunity involves processes such as phagocytosis, encapsulation, and nodulation (Coates *et al*., 2022). Phagocytosis is the process of recognition, binding, and ingesting pathogens, especially bacteria. Nodulation involves the aggregation of hemocytes around pathogens, thereby entrapping large numbers of bacteria, and is particularly useful in dealing with greater bacterial loads. Encapsulation is the process of capsule formation around larger pathogens such as parasites, protozoa, and nematodes.

Humoral immunity involves melanisation (Fig. 1B), antimicrobial peptide (AMP) production *via* the Toll pathway (Fig. 1C), the immune deficiency (IMD) pathway (Fig. 1D), the Janus kinase/signal transducers and activators of transcription (JAK–STAT) pathway (Fig. 1E) and antiviral responses *via* the RNA interference (RNAi) pathway (Fig. 1F). Unlike cellular immunity, which is carried out by immune cells constitutively present in the body, the humoral immune response is induced by pathogens. Melanisation is initiated when pathogen particles such as lipopolysaccharides (LPSs) and peptidoglycan (PGN) bind with soluble pathogen recognition receptors (PRRs) and stimulate a serine protease cascade (Nakhleh, El Moussawi & Osta, 2017). This serine protease produces prophenoloxidase‐activating proteinase (PAP) from proPAP (Fig. 1B). PAP then activates conversion of prophenoloxidase (proPO) to phenoloxidase (PO). PO converts phenol to quinones which ultimately produce melanin through a series of biomolecular conversions. Melanin surrounds and eliminates pathogens through exposure to reactive oxygen species (ROS) and lysis from toxic melanin intermediates. In addition, melanin neutralises pathogens by contributing to nodulation and encapsulation which involves melanin accumulation and haemocyte aggregation around pathogens.

The Toll pathway begins with the recognition of Gram‐positive bacterial PGN, lysine‐type peptidoglycan (LYS‐PGN), by the soluble peptidoglycan receptors PGRP‐SA and PGRP‐SD. This receptor–ligand binding stimulates the proteolytic cleavage of Spätzle protein which then binds with surface protein Toll‐like receptors (TLRs), and activates the Toll signalling pathway (Fig. 1C). The IMD pathway is activated when the Gram‐negative bacterial PGN, diaminopimelic acid‐containing peptidoglycan (DAP‐PGN), binds with the cell surface receptor PGRP‐LC (Fig. 1D). The JAK–STAT pathway is activated when unpaired (UPD) binds with surface protein Domeless (Dome) (Fig. 1E) (Myllymäki & Rämet, 2014). Activation of Toll, IMD and JAK–STAT pathways leads to the induction of the transcription factors dorsal‐related immune factor (DIF), Relish and STAT dimer, respectively, which then translate sets of target AMPs (Lemaitre & Hoffmann, 2007). Many AMPs kill bacteria by disrupting bacterial cell membranes, but they can also inhibit cellular functions such as enzymatic activity, or protein or nucleic acid synthesis (Zhang & Gallo, 2016).

The RNAi pathway is initiated when viral double‐stranded RNA (dsRNA) is recognised by the RNase‐III enzyme Dicer‐2 (Meister & Tuschl, 2004). Dicer‐2 cleaves dsRNA into approximately 21‐nucleotide small interfering RNA (siRNA) duplexes. Each siRNA duplex is later incorporated into the RNA‐induced silencing complex (RISC) and viral RNA is degraded by RISC (Meister & Tuschl, 2004) (Fig. 1F).

Recent studies have highlighted that the insect microbiome also functions to protect insects from infection, by (*i*) stimulating the insect immune system, (*ii*) producing toxins that kill pathogens directly and (*iii*) reducing the levels of nutrients available for the pathogen (Carrington *et al*., 2018; Daisley *et al*., 2020a; Lang *et al*., 2021; Miller, Smith & Newton, 2021; Janke *et al*., 2022). In this review, we include the protective effects of the insect‐associated microbiota as a component of the insect immune system (Fig. 1A).

## III. IMMUNE RESPONSE, PATHOGEN RESISTANCE, AND FITNESS

Insect immune responses (Fig. 1) involve the expression of effectors, such as AMPs, or the activation of enzymes, such as PO, to sequester and reduce the spread of pathogens across the body, inhibit their growth, or directly kill them. Also, hosts can avoid infection or reduce infection risk by behavioural strategies (Gibson & Amoroso, 2022; Milutinović & Schmitt, 2022). In the studies reviewed here, insect immune responses are often measured by the total number of immune cells (i.e. phagocytes) present, by the activity of enzymes involved in the melanisation pathway (e.g. PO), by the expression of genes for components involved in melanisation (e.g. proPO and PO), by levels of components of immune activation pathways (e.g. cactus, relish) or by the expression of AMP genes (e.g. *defensin, attacin*). Newer studies use more integrative approaches such as transcriptomics or proteomics to capture a significant proportion of immune system activation. Each measurement has benefits and drawbacks, and method selection often depends on the research question (Boughton, Joop & Armitage, 2011).

Pathogen resistance is the ability of an insect host to limit pathogen growth, thereby reducing pathogen‐mediated fitness costs. Pathogen resistance largely depends on host immune defences but also is related to the pathogen's ability to circumvent the insect immune response. Pathogen resistance is often measured by the pathogen load in the host or by the survival of the host and/or the pathogen. Pathogen tolerance is the ability of the host to maintain fitness despite being infected and is predicted to have a neutral or positive effect on pathogen fitness (Kutzer & Armitage, 2016). Pathogen tolerance is measured by quantifying fitness indices (e.g. growth, survival, reproduction) of both host and pathogen.

Insect immune responses are essential for pathogen resistance, however, an induced immune response involving non‐specific changes such as elevated PO levels and hemocyte numbers, does not necessarily confer pathogen resistance (Adamo & Lovett, 2011; Silva & Elliot, 2016; Mastore *et al*., 2019). Induced immune responses can also fail to ensure pathogen resistance when they are insufficient, such as a weak AMP or melanisation response, or because pathogens have evolved mechanisms to circumvent host defences (Vallet‐Gely, Lemaitre & Boccard, 2008). Often immune responses are elicited experimentally, yet whether such stimulation generates useful resistance depends on the pathogens studied and therefore is of limited general applicability (Adamo & Lovett, 2011).

Initiating and maintaining an immune response is energy demanding (Moret & Schmid‐Hempel, 2000; Schmid‐Hempel, 2005). For instance, antimicrobial effectors are often proteins or peptides and their expression depletes the insect's amino acid reservoirs, and enzyme activity requires ATP and therefore depletes energy reserves. Because insects have a limited supply of food and energy, they are likely to trade off immune responses against other physiological process such as growth, development and reproduction (Rolff & Siva‐Jothy, 2002; Adamo & Lovett, 2011; Bajgar *et al*., 2015). Activating and maintaining immune responses is essential for protection against pathogens. However, persistent activation of immune responses by non‐pathogenic entities such as microplastics or pharmaceuticals could reduce insect fitness by depleting energy reserves and thereby reducing reproductive output. Furthermore, activation of immune responses, such as the PO cascade, can result in damage to insect tissues or organs, again reducing fitness (Khan, Agashe & Rolff, 2017). Because the activation of immune responses does not necessarily guarantee pathogen resistance, and given that such activation is costly in terms of energy expenditure and immunopathology, an activated immune response is unlikely to be beneficial in an uninfected host, unless it reduces pathogen‐driven fitness costs in infected hosts.

## IV. ECOLOGICAL FACTORS IMPACT INSECT IMMUNITY

Insect immunity is a plastic trait that is shaped by the host genome, physiological conditions and local environment (Rolff & Siva‐Jothy, 2003). Pathogens are one of the strongest selective agents that shape immunity (Horrocks, Matson & Tieleman, 2011; Vogelweith *et al*., 2017). Pathogen exposure varies among species according to habitat, diet and behaviour (Siva‐Jothy, Moret & Rolff, 2005). Similarly, within a species, pathogen infection and immune response can vary with sex, developmental stage and social caste (Rolff, 2001; Leech *et al*., 2019; Paul, Khan & Herberstein, 2022). Variation in insect immune defences within and among species is therefore shaped by differential pressures and likely also by risk of wounding (Subasi *et al*., 2024). The expression of immune traits, like many other physiological traits, is energy dependent. Therefore, ecological factors that impact resource availability, energy reserves, nutritional status and life‐history traits also impact insect immunity (Rolff & Siva‐Jothy, 2003; Horrocks *et al*., 2011; Vogelweith *et al*., 2017).

In addition to abiotic factors, biotic factors such as population density, and inter‐ and intraspecific interactions can affect insect immunity (McNamara, Wedell & Simmons, 2013; Cinel, Hahn & Kawahara, 2020; Green, 2021). At higher population densities some insects exhibit density‐dependent prophylaxis and invest more into immune function due to the resulting increased risk of infection (Green, 2021). In addition, sexual selection and male–male competition for mating can also influence investment into immune responses (McNamara *et al*., 2013). Interspecific interactions such as competition for shared resources and predator‐induced stress can result in reduced immune function and increased infection rates (Joop & Rolff, 2004; Cinel *et al*., 2020; Sun & Bai, 2020).

## V. ANTHROPOGENIC FACTORS IMPACTING INSECT IMMUNITY

**Fig. 2:**
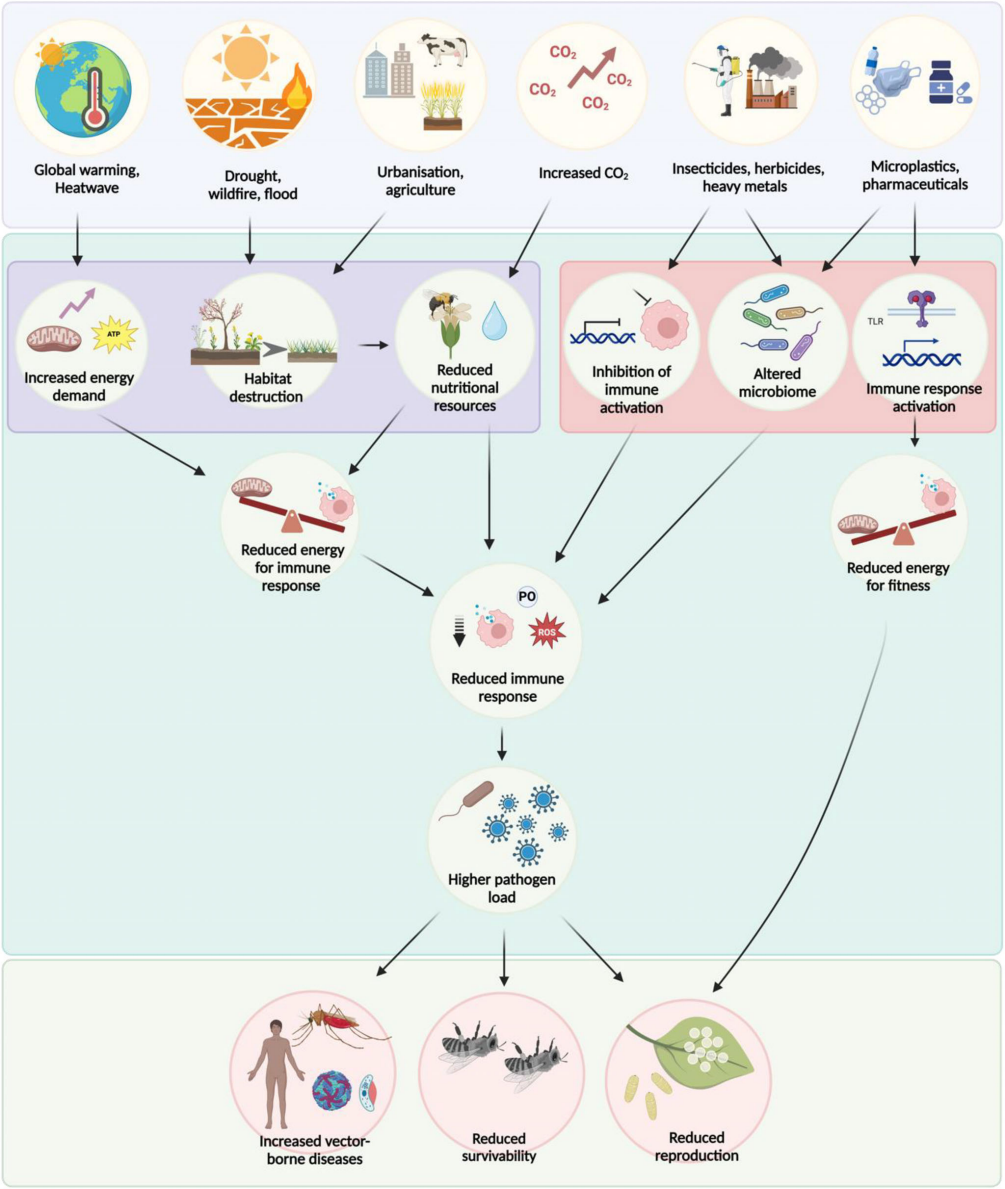
Summary of routes by which anthropogenic factors impact insect immune responses to pathogen exposure. At elevated temperatures, energy demands increase in insects to meet physiological demands thereby reducing energy availability for induction of immune responses. Extreme climatic events such as flood, wildfire, and drought, climate change variables such as elevated CO_2_ levels, urbanisation and intensive agriculture practices, can all reduce food availability and consequently reduce the energy available for immune responses. Insecticides, herbicides and heavy metals can directly inhibit insect immune activation pathways. As a consequence, these anthropogenic factors reduce the insect's ability to induce immune responses and eliminate pathogens upon infection. Most anthropogenic factors promote a higher pathogen load and, therefore, reduce insect fitness by causing greater pathogen‐driven mortality and reduced reproductive effort as well as increasing transmission of vector‐borne diseases. Some anthropogenic factors such as microplastics and some pharmaceuticals reduce insect fitness by activating the insect immune system, depleting energy reserves and reducing investment into fitness traits such as reproduction. Figure created with BioRender.com.

Aspects of anthropogenic global change, such as anthropogenic climate change (increasing temperature and frequency of extreme climatic events such as fire, heatwaves, drought and flood), urbanisation‐related land cover, intensive agricultural practices, and pollution (insecticides, herbicides, heavy metals, pharmaceuticals), can alter insect immune responses and pathogen defence, resulting in reduced fitness in terms of both survival and reproduction (Table 1; Fig. 2). Pollution with microplastics, pesticides, heavy metals and pharmaceuticals impact insect immunity directly by impairing immune activation. Other anthropogenic factors including elevated temperatures increase energy demand while elevated CO_2_ alters the quality and availability of dietary resources, both of which consequently reduce resources available for immune responses. In this section we provide examples of studies that investigated direct and indirect (global warming, landscape change, extreme climatic events) consequences of anthropogenic factors on insect immune responses and how altered immune responses impact pathogen resistance and infection outcome.

**Table 1:** Reported impacts of anthropogenic factors on insect immunity and host–pathogen interactions.

Anthropogenic factor	Anthropogenic stressors	Species	Order	Impact on immune response	Impact on host–pathogen interactions	Reference
Climate change	Temperature	*Lobesia botrana*	Lepidoptera	Moths reared at higher temperature (28 °C) showed reduced phenoloxidase (PO) activity and lower hemocyte count compared to those reared at 22 and 25 °C	NA	Iltis *et al*. (2018)
	Temperature	*Anticarsia gemmatalis*	Lepidoptera	Capsule melanisation was greater in larvae reared at higher temperature (32 °C) compared to (26, 28 and 30 °C)	Caterpillars reared at higher temperature were killed by multiple nucleopolyhedrovirus (AgMNPV) more rapidly than caterpillars reared at lower temperatures	Silva & Elliot (2016)
	Temperature	*Sarcophaga africa*	Diptera	Larvae reared at 30 °C showed increased PO but not lysozyme activity compared to those reared at 10 and 20 °C	Larvae reared at 30 °C showed increased susceptibility to *Steinernema feltiae*, *S. carpocapsae* and *Heterorhabditis bacteriophora,* but reduced susceptibility to *Bacillus thuringiensis* compared to those reared at 10 and 20 °C	Mastore *et al*. (2019)
	Temperature	*Galleria mellonella*	Lepidoptera	Larvae reared at 30 °C showed increased PO and lysozyme activity compared with larvae reared at 10 and 20 °C	Larvae reared at 30 °C showed increased susceptibility to *Steinernema carpocapsae* and *Heterorhabditis bacteriophora,* but reduced susceptibility to *Steinernema feltiae* and *Bacillus thuringiensis* compared with larvae reared at 10 and 20 °C	Mastore *et al*. (2019)
	Temperature	*Melanoplus sanguinipes*	Orthoptera	Grasshoppers reared at 39 °C had reduce PO titre compared to those reared at 27 °C	Grasshoppers reared at 39 °C showed greater susceptibility to *Beauveria bassiana* fungal infection compared to those reared at 27 °C	Srygley & Jaronski (2022)
	Temperature	*Drosophila melanogaster*	Diptera	NA	Flies reared at higher (28 °C) compared to lower (23 and 18 °C) temperature exhibited higher bacterial load and mortality when infected with *Providencia rettgeri*	Lazzaro *et al*. (2008)
	Temperature	*Drosophila melanogaster*	Diptera	NA	Flies reared at higher (28.5 °C) compared to lower (21.5 °C) temperature exhibited higher percentage mortality when infected with *Pseudomonas aeruginosa*	Kutch *et al*. (2014)
	Temperature	*Pieris napi*	Lepidoptera	Butterflies reared at 25 °C had lower hemocyte number and proPO activity compared to butterflies reared at 17 °C	NA	Bauerfeind & Fischer (2014)
	Temperature	*Tenebrio molior*	Coleoptera	Beetle larvae reared at 30 °C showed reduced PO and lysozyme activities compared to beetles reared at 18, 22, and 26 °C.	Beetle larvae reared at 30 °C showed reduced antibacterial performance compared to those reared at 18, 22, and 26 °C.	Tao *et al*. (2014)
	Temperature	*Culex pipiens*	Diptera	NA	Mosquitoes incubated at 30 °C had higher infection rates compared with mosquitoes incubated at 18, 20 and 26 °C when infected with West Nile Virus	Dohm *et al*. (2002)
	Temperature	*Aedes aegypti*	Diptera	Highest number of immune genes upregulated in Chikungunya virus‐infected mosquitoes when kept at 18 and 28 °C but not at 32 °C	Chikungunya virus levels were higher when infected mosquitoes were kept at 32 °C compared to 18 and 28 °C	Wimalasiri‐Yapa *et al*. (2021)
	Temperature	*Aedes japonicus*	Diptera	NA	Zika virus‐infected *Aedes japonicus* showed greater infection rate and higher viral titre when incubated at 27 °C compared to 21 °C	Jansen *et al*. (2018)
	Heatwave	*Bicyclus anynana*	Lepidoptera	PO activity and hemocyte number were lower in butterflies exposed to 34 °C for 24 h compared to those exposed to 10 and 27 °C	NA	Karl *et al*. (2011)
	Heatwave	*Gryllus texensis*	Orthoptera	Elevated temperature (reproducing a heatwave) of 33 °C for 6 days (28% of lifespan) increased PO and lysozyme activity	Compared to crickets exposed to 18 °C, those exposed to an elevated temperature (33 °C) showed increased % mortality when infected with *Bacillus cereus* but showed reduced % mortality when infected with *Serratia marcescens*	Adamo & Lovett (2011)
	Heatwave	*Coenagrion puella*	Odonata	Simulated heatwave (26 °C for one day followed by 32 °C for three days) increased encapsulation but reduced growth in heatwave‐exposed damselfly larvae	Larvae exposed to simulated heatwave (26 °C for one day followed by 32 °C for three days) showed higher mortality compared to larvae kept at 21 °C	Tüzün & Stoks (2021)
	Temperature fluctuations	*Lycaena tityrus*	Lepidoptera	Hemocyte numbers were greater in larvae reared under fluctuating temperatures (either 14–24 °C or 20–30 °C) compared to larvae reared at constant temperature (17.7 °C or 23.7 °C)	NA	Fischer *et al*. (2011)
	Drought	*Melitaea cinxia*	Lepidoptera	Mild drought upregulated expression of the gene *pelle*, but not PO activity or other immune genes	NA	Rosa *et al*. (2019)
	Drought	*Tomicus piniperda*	*Coleoptera*	NA	Drought‐exposed beetles showed greater resistance against entomopathogenic fungus *Beauveria bassiana*	Krams *et al*. (2012)
	Elevated CO_2_	*Danaus plexippus*	Lepidoptera	NA	Monarch butterflies reared on host plants grown in 760 ppm CO_2_ showed reduced tolerance to *Ophryocystis elektroscirrha* infection. Elevated CO_2_ also increased parasite virulence	Decker *et al*. (2018)
	Elevated CO_2_	*Danaus plexippus*	Lepidoptera	Uninfected monarch butterflies showed reduced PO activity, and hemocyte and granulocyte concentration (cells/μl) when reared on milkweed grown at 810 ppm CO_2_ compared to when reared on milkweed grown at 410 ppm CO_2_.	NA	Decker *et al*. (2021)
	Elevated CO_2_	*Paropsis atomaria*	Coleoptera	Beetles feeding on *Eucalyptus tereticornis* seedlings grown under elevated CO_2_ (640 ppm) showed increased melanisation but reduced PO activity and haemolymph protein concentrations	NA	Gherlenda *et al*. (2016)
	Elevated CO_2_	*Helicoverpa armigera*	Lepidoptera	Larvae raised on wheat grown in elevated CO_2_ (750 ppm) and parasitised with *Microplitis mediator* showed reduced total hemocyte count, impaired hemocyte spreading capacity and reduced encapsulation ratio compared to when reared on wheat grown at ambient CO_2_ (375 ppm)	NA	Yin *et al*. (2014)
Landscape change	Agriculture	*Apis melifera*	Hymenoptera	Bees from colonies surrounded by primarily non‐agricultural forage had greater expression of immune genes for abaecin, apidaecin, defensin 2, and lysozyme 2 compared to bees from colonies surrounded by more intensively cultivated agricultural lands	NA	Ricigliano *et al*. (2019)
	Urbanisation	*Apis mellifera*	Hymenoptera	Feral colonies have lower disease burdens and stronger immune responses compared to managed bees	Pathogen pressure increases and survival declines along a gradient of increasing urbanisation	Youngsteadt *et al*. (2015)
	Landscape change	*Bombus impatiens*	Hymenoptera	NA	Bumble bees in habitat with sparse floral and nesting resources had higher pathogen load	McNeil *et al*. (2020)
Pollution	Cd	*Lymantria dispar*	Lepidoptera	Cd stress reduced total hemocyte numbers, phagocytosis activity and encapsulation, and reduced expression of the antimicrobial peptides (AMPs) cecropin and lebocin	NA	Wu *et al*. (2022)
	Cu	*Protophormia terraenovae*	*Diptera*	Flies reared on Cu‐contaminated food showed increased encapsulation compared to those reared on uncontaminated food	NA	Pölkki *et al*. (2012)
	Ni	*Galleria mellonella*	Lepidoptera	NA	Larvae infected with the fungus *Beauveria bassiana* fed on a diet containing Ni showed greater mortality compared to infected larvae fed a control diet	Dubovskiy *et al*. (2011)
Insecticides	Permethrin	*Anopheles gambiae*	Diptera	Sublethal permethrin exposure increased melanisation in individually reared mosquito larvae but not in larvae when reared in groups	Sublethal permethrin exposure reduced bacterial load in individually reared mosquito larvae but not in larvae reared in groups	Hauser & Koella (2020)
Insecticides	Imidacloprid	*Drosophila melanogaster*	Diptera	Imidacloprid dysregulated the IMD pathway which consequently inhibited Duox expression and reduced H_2_O_2_ production	NA	Chmiel *et al*. (2019)
	Imidacloprid	*Apis mellifera*	Hymenoptera	Bee larvae exposed to field‐realistic concentration of imidacloprid, showed reduced expression of the AMPs abaecin, apidaecin, apisimin and hymenopteacin	NA	Wu *et al*. (2017)
	Imidacloprid	*Apis mellifera*	Hymenoptera	NA	Sublethal doses of Imidacloprid increased the load of the gut parasites *Nosema ceranae* and *Nosema apis*	Pettis *et al*. (2012)
	Flupyradifurone	*Apis mellifera*	Hymenoptera	Sublethal pesticide exposure reduced expression of defensin‐1	Sublethal doses of flupyradifurone increased the load of the gut parasite *Nosema ceranae*	Al Naggar & Baer (2019)
	Thiamethoxam	*Apis mellifera*	Hymenoptera	NA	Sublethal dose of thiamethoxam reduced survival when infected with *Bacillus badius* and *Ochrobactrum anthropic*	Decio *et al*. (2021)
	Imidacloprid	*Nannotrigona perilampoides*	Hymenoptera	Exposure to field‐realistic concentration of imidacloprid reduced expression of the AMPs abaecin, defensin1, and hymenopteacin	NA	Al Naggar *et al*. (2022a)
	Thiacloprid, imidacloprid, and clothianidin	*Apis mellifera*	Hymenoptera	Exposure to these neonicotinoids reduced total hemocyte number, encapsulation response, and antimicrobial activity	NA	Brandt *et al*. (2016)
	Clothianidin	*Apis mellifera*	Hymenoptera	NA	Clothianidin inhibited nuclear factor kappa B (NF‐κB) immune signalling pathway and consequently promoted replication of a viral pathogen, deformed wing virus (DWV), in honey bees	Di Prisco *et al*. (2013)
	Imidacloprid	*Apis mellifera*	Hymenoptera	Chronic exposure to sublethal doses of imidacloprid decreased vitellogenin transcript levels in *Nosema ceranae*‐infected bees	Chronic exposure to sublethal doses of imidacloprid increased DWV infection in *Nosema ceranae*‐infected bees and reduced survival	Balbuena *et al*. (2023)
	Flupyradifurone, sulfoxaflor, and azoxystrobin	*Apis mellifera*	Hymenoptera	NA	Chronic exposure to these insecticides and fungicides reduced diversity of gut microbiota and increased relative abundance of the pathogenic bacterium *Serratia marcescens*	Al Naggar *et al*. (2022b)
Fungicides	Captan and difenoconazole	*Apis mellifera*	Hymenoptera	Difenoconazole treatment decreased granulocyte numbers 1 h after treatment, followed by an increase 24 h after treatment	NA	Kaur *et al*. (2023)
	Boscalid and pyraclostrobin	*Apis mellifera*	Hymenoptera	NA	Bees fed fungicide‐treated pollen had higher titres of deformed wing virus and black queen cell virus	Degrandi‐Hoffman *et al*. (2015)
	Chlorothalonil	*Apis mellifera*	Hymenoptera	Reduced expression of immune deficiency (*imd*) gene were detected in chlorothalonil‐fed honey bees	NA	Conradie *et al*. (2024)
	Pyraclostrobin	*Apis mellifera*	Hymenoptera	Pyraclostrobin induced expression of defensin1, and hymenoptaecin, and decreased expression of apidaecin, and abaecin in larvae. In pupae, pyraclostrobin increased expression of apidaecin, and hymenoptaecin, and decreased expression of defensin1	NA	Xiong *et al*. (2023)
Herbicides	Glyphosate	*Culex pipiens*	Diptera	NA	Exposure to field‐realistic doses of glyphosate increased prevalence of malaria parasite when larvae were reared in nutritional stress conditions compared to standard diet	Bataillard *et al*. (2020)
Herbicides	Glyphosate	*Apis mellifera*	Hymenoptera	Glyphosate treatment decreased expression of the AMPs apidaecin, defensin and hymenoptaecin, and reduced melanisation in bee hemolymph	NA	Motta *et al*. (2022)
	Glyphosate	*Anopheles gambiae*	Diptera	NA	Glyphosate increased the abundance of the malaria‐causing parasite *Plasmodium falciparum* in mosquitoes	Smith *et al*. (2021)
Herbicides	Glyphosate	*Galleria mellonella*	Lepidoptera	Glyphosate decreased the size of melanised nodules in hemolymph	Glyphosate reduced survival of caterpillars infected with the fungus *Cryptococcus neoformans*	Smith *et al*. (2021)
	Pendimethalin‐based herbicide	*Harpalus rufipes*	Coleoptera	Herbicides reduced numbers of circulating haemocytes and phagocytic index in beetles	NA	Vommaro *et al*. (2021)
	Glyphosate‐based herbicides	*Apis mellifera*	Hymenoptera	NA	Feeding on glyphosate‐based herbicides reduced survival in bees infected with *Nosema* fungus	Faita *et al*. (2020)
	Glyphosate‐based herbicides	*Apis mellifera*	Hymenoptera	Glyphosate treatment decreased the abundance of the symbiotic bacteria *Snodgrassella alvi*	Bees infected with *Serratia marcescens* exhibited increased mortality in hives exposed to glyphosate compared to control hives	Motta *et al*. (2020)
Pharmaceuticals	Oxytetracycline	*Apis mellifera*	Hymenoptera	Oxytetracycline treatment depleted immune regulatory symbionts *Frischella perrera* and *Lactobacillus* Firm‐5 strains.	Oxytetracycline treatment reduced antimicrobial capacity of adult hemolymph against *Arthrobacter globiformis* by 31.27%.	Daisley *et al*. (2020b)
	Tetracycline	*Apis melifera* and *A. cerana*	Hymenoptera	NA	In *A. cerana* but not *A. mellifera*, tetracycline treatment increased titres of Israeli acute paralysis virus (IAPV) and reduced survival due to IAPV infection	Deng *et al*. (2022)
	Tetracycline	*Apis mellifera*	Hymenoptera	Tetracycline exposure reduced abundance of the symbionts *Lactobacillus* Firm‐5, *Lactobacillus* Firm‐4 and *Snodgrassella alvi*	Tetracycline exposure reduced survival of bees when challenged with the pathogenic bacterium *Serratia* kz11	Raymann *et al*. (2017)
	Tylosin	*Apis melifera*	Hymenoptera	Reduced expression of the AMPs apidaecin, and defensin‐2 were detected in tylosin‐exposed honey bees.	NA	Motta *et al*. (2022)
	Ibuprofen	*Chironomus riparius*	Diptera	Exposure to ibuprofen (0.01, 1, and 100 μg/L) increased expression of immune genes proPO and defensin	NA	Muñiz‐González (2021)
	Ivermectin	*Scathophaga stercoraria*	Diptera	Exposure to the ivermectin elevated basal PO activity	NA	West & Tracy (2009)
Microplastics	Polyethylene	*Chironomus riparius*	Diptera	Ingestion of polyethylene microplastic increased basal PO activity in aquatic larval stage	NA	Silva *et al*. (2021a)
	Polystyrene	*Bombyx mori*	Hymenoptera	Polystyrene microplastic exposure increased expression of lysozyme and cecropin	NA	Muhammad *et al*. (2021)
	Polystyrene	*Apis melifera* and *Apis cerana*	Hymenoptera	NA	Bees infected with Israeli acute paralysis virus and exposed to microplastic had higher viral load and exhibited greater mortality compared to bees that were not exposed to microplastic	Deng *et al*. (2021)

### (1) Anthropogenic climate change

#### (a) Global warming

Greenhouse gas emissions caused by burning fossil fuels and agriculture are causing global warming and it is predicted that by 2100 global temperatures will rise between 2.2 and 3.5 °C compared to 1850–1900 (Lee *et al*., 2023). The influence of temperature on insect immunity has been studied in species from different ecological regions. For example, gall thrips (*Gynaikothrips uzeli*) from different latitudes along the east coast of China showed increases in PO activity and expression of eight Toll pathway immune genes with increasing latitude and decreasing temperature (Yu *et al*., 2020). Thrips from higher latitudes were better protected from *Beauveria bassiana* fungal infection compared to those from lower latitudes (Yu *et al*., 2020). A similar pattern of greater immune responses in cooler regions was found in two species of rolled‐leaf beetles (Chrysomelidae) at two different altitudes in Costa Rica. Beetles from a higher altitude (2000 m) and lower temperatures (10–20 °C) showed a greater melanisation response than beetles from a lower altitude (50 m) with higher temperatures (20–32 °C) (González‐Tokman *et al*., 2019).

**Fig. 3:**
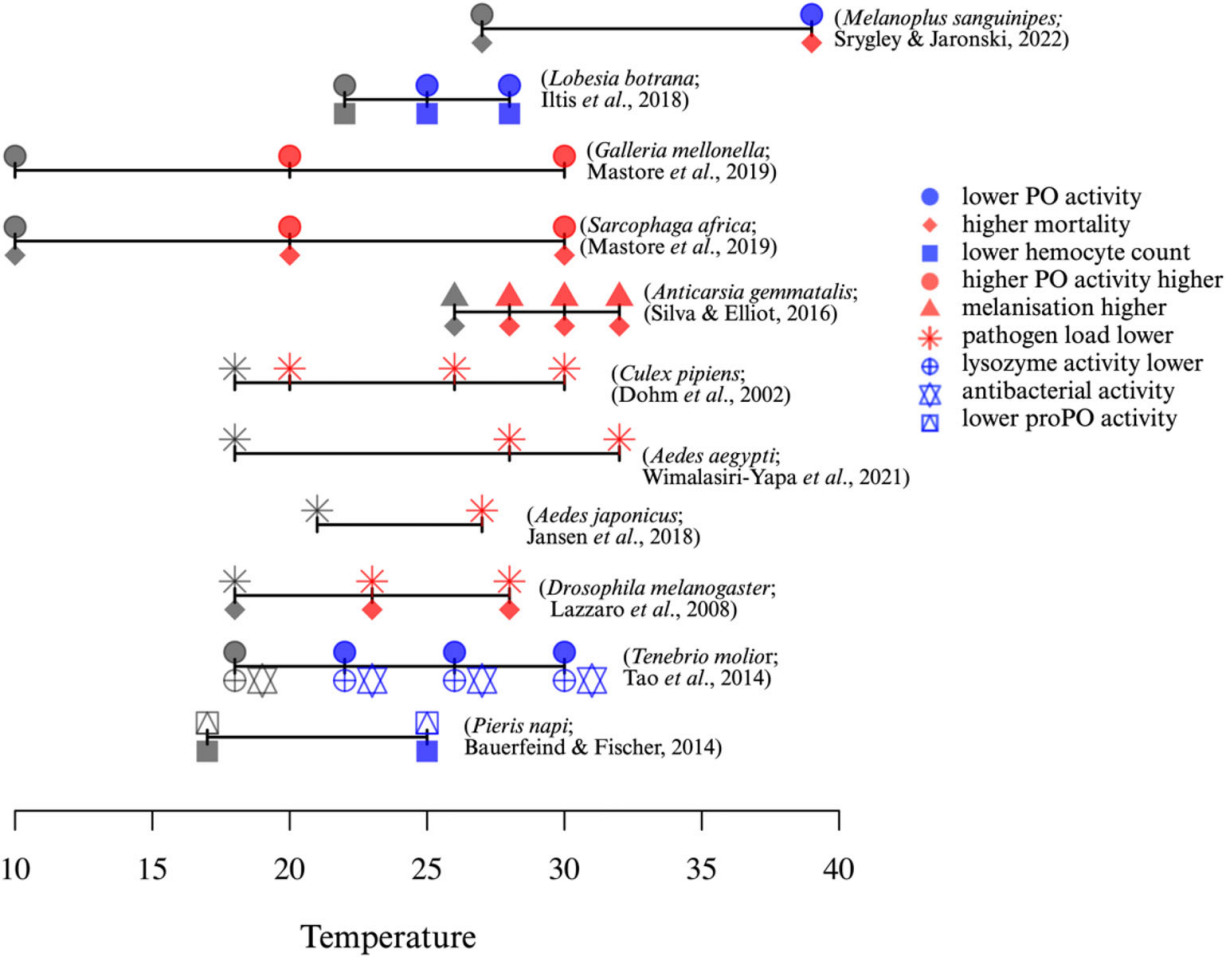
Selected temperature effects on immune defence in insects. The horizontal bars show the range of temperatures examined in the cited studies. Shape and colour of the pictograms show study outcome as described in the key. The lowest experimental temperature is shown in grey and used as a control. The measured defence parameter (e.g. immune response, pathogen load) is shown in blue when it had a lower value compared to the control, or red when the value was higher. PO, phenoloxidase; proPO, prophenoloxidase. Figure created in R version 4.2.1 (R Core Team, 2022).

In controlled laboratory experiments, rearing temperatures have been shown to change immune responses and resistance against pathogens (Fig. 3, Table 1). In the European grapevine moth, *Lobesia botrana*, a higher rearing temperature (28 °C) was correlated with reduced PO activity and lower hemocyte counts than when reared at 25 °C and 22 °C (Iltis *et al*., 2018). Similarly, *Melanoplus sanguinipes* grasshoppers reared at 39 °C had a lower PO titre compared to grasshoppers reared at 27 °C both before and after *B. bassiana* fungal infection (Srygley & Jaronski, 2022). The reduced immune response at higher temperature also increased susceptibility to *B. bassiana* fungal infection; cumulative death after 2 weeks of infection in grasshoppers reared at 39 °C was 100% compared to 85% in crickets reared at 27 °C (Srygley & Jaronski, 2022). At higher temperatures insects may trade off investment in immunity with other physiological processes resulting in greater pathogen‐driven mortality following infection (Fig. 2). In the majority of the studies cited in Fig. 3, increasing temperature had a detrimental effect on insect immune function and/or resistance and survival, but these impacts are likely to depend on habitat and may vary across a species' range.

In contrast to the previously cited studies, the greater wax moth *Galleria mellonella* and the fly *Sarcophaga africa* showed greater PO and lysozyme activity when larvae were reared at 30 °C compared to larvae reared at 10 and 20 °C (Mastore *et al*., 2019). *G. mellonella* are adapted to live in warm bee hives, therefore a temperature of 30 °C may be more optimal for their physiological performance compared to 10 and 20 °C, probably explaining their greater immune response at 30 °C. The greater immune response in *G. mellonella* at the higher temperature (30 °C) reduced mortality following infection with the nematode *Steinernema feltiae* and the bacterium *Bacillus thuringiensis* but increased mortality when infected with the nematodes *Steinernema carpocapsae* and *Heterorhabditis bacteriophora* (Mastore *et al*., 2019), suggesting that induced immune responses at higher temperatures may provide protection against some pathogens but may reduce immunocompetence against others. In *Sarcophaga africa*, infection with the nematodes *S. feltiae*, *S. carpocapsae* and *H. bacteriophora* caused greater mortality at 30 °C compared to 10 and 20 °C even though immune responses were greater at 30 °C (Mastore *et al*., 2019). Similarly, caterpillars of the moth *Anticarsia gemmatalis* showed a greater immune response (capsule melanisation) when reared at higher temperature (32 °C), however, these caterpillars succumbed to a nucleopolyhedrovirus (AgMNPV) infection more rapidly than caterpillars reared at lower temperatures (26, 28, 30 °C) (Silva & Elliot, 2016). Higher mortality upon infection at higher temperatures may occur in insects even when they have a greater immune response because temperature‐mediated activation of a non‐specific immune response does not provide protection against all pathogens. For example, a temperature‐mediated increase in melanisation may provide protection against bacteria and parasites but not against viruses (Silva & Elliot, 2016). Alternatively, the strength of the insect immune response and the growth and virulence of the pathogen may change at different rates with different temperatures (Linder, Owers & Promislow, 2008; Apidianakis & Rahme, 2009). For example, *Gryllus texensis* crickets showed increased PO and lysozyme activity when kept at 33 °C for 6 days (28% of their lifespan) compared to those kept at 18 and 26 °C (Adamo & Lovett, 2011). The pathogen *Bacillus cereus* also reproduced more rapidly when incubated at 33 °C compared to 18 and 26 °C (Adamo & Lovett, 2011). Despite their greater immune response, mortality of *B. cereus*‐infected crickets was greater at 33 °C compared to 18 and 26 °C (Adamo & Lovett, 2011), presumably due to the rise in pathogen virulence overcoming the improved immune response of the crickets (Adamo & Lovett, 2011). Overall, while a higher rearing temperature can sometimes improve insect immune responses, this does not necessarily translate into resistance against pathogens (Fig. 3) and may reduce fitness *via* immunopathology and by reducing energy availability for reproduction (Fig. 2).

#### (b) Temperature fluctuations and heatwaves

As a consequence of climate change, extreme summer heatwaves are becoming more frequent and severe (Stillman, 2019). Heatwaves and rapid temperature fluctuations both impact insect physiology. For example, in the butterfly *Lycaena tityrus* hemocyte numbers were greater when reared at fluctuating temperatures (either 14–24 or 20–30 °C) than when reared at a constant temperature (17.7 or 23.7 °C) (Fischer *et al*., 2011). In the damselfly *Coenagrion puella* heatwaves increased immune responsiveness (encapsulation) but this showed a trade off with reduced growth (Tüzün & Stoks, 2021). *C. puella* damselfly larvae exposed to experimental heatwaves for 4 days (1 day at 26 °C followed by 3 days at 32 °C) consumed more energy and had less energy available for other physiological functions compared to control larvae that experienced a constant temperature of 21 °C (Tüzün & Stoks, 2021). As a consequence, despite having stronger immune responses, immune‐challenged larvae that experienced a simulated heatwave showed higher mortality compared to larvae that did not experience heatwaves (Tüzün & Stoks, 2021).

The impact of temperature fluctuations also depends on the local climate, temperatures experienced during development, and the extent of temperature fluctuations. For instance, *Ischnura elegans* damselflies from high latitudes (mean annual temperature, 20 °C) when reared at an elevated temperature (24 °C) showed reduced PO activity when exposed to higher daily temperature fluctuations (10 °C) compared to fluctuations of 5 or 0 °C (Verheyen & Stoks, 2019). However, low‐latitude larvae (mean annual temperature 24 °C) reared at their native temperature (24 °C) showed increased PO activity at higher daily fluctuations (10 °C) compared to no daily fluctuations (Verheyen & Stoks, 2019). Therefore, the combination of rearing temperature and the extent of temperature fluctuations needs to be considered when interpreting the impact of anthropogenic climate change on an insect's immunity.

#### (c) Drought and wildfire

Climate change is likely to result in increased frequencies of extreme events such as drought, flood and wildfire (Frame *et al*., 2020). These extreme climatic events can directly cause mortality but also indirectly impact insect physiology and insect–pathogen interactions by fragmenting habitats (Filazzola, Matter & MacIvor, 2021).

Drought may have different impacts on different insect groups, for example phytophagous insects may respond to drought quite differently compared to aquatic insects. Mild drought may increase levels of amino acids and sugars in plant leaves, thereby benefiting the nutrition and immune responses of phytophagous insects (Krasensky & Jonak, 2012). For instance, *Melitaea cinxia* butterflies raised on moderately water‐stressed plants showed increased expression of the Toll pathway gene *pelle*, but no such effect was reported for PO activity or other immune genes (Rosa *et al*., 2019). Whether the increased expression of *pelle* leads to greater production of AMPs *via* the Toll pathway and ultimately increased resistance in infected butterflies has not been tested. On the other hand, drought can negatively impact wetland areas, reducing the availability of food resources for aquatic insects which can reduce investment in immunity and pathogen resistance (Fig. 2). A common garden study on *Aedes aegypti* mosquito lines from an arid region in Senegal showed increased susceptibility to Zika virus infection compared to a mosquito line from a less arid region (Accoti *et al*., 2023). Five days after a blood meal, the 50% oral infectious dose (OID_50_) of the Zika virus was approximately three times higher in the mosquito line from the arid region [7.19 log_10_ focus‐forming units (FFU)/mL] compared to the mosquito line from the less‐arid region (2.25 log_10_ FFU/mL) (Accoti *et al*., 2023).

Wildfires alter landscape composition, reducing total food availability and diversity of food resources, which both contribute to reduced energy availability for investment into immunity, and may thereby alter host–pathogen interactions (Fig. 2). Furthermore, wildfire ash contains polycyclic aromatic hydrocarbons and heavy metal pollutants such as Co, Ni, Cu, Zn, As, Cd, Pb and Hg (Harper *et al*., 2019) which can directly impair insect immunity (Muñiz‐González, 2021). In addition, poor air quality and high temperature in wildfire‐prone areas was associated with reduced gene expression of proPO in *Apis mellifera* (Mayack *et al*., 2023).

#### (d) Elevated CO_2_ levels

Atmospheric CO_2_ concentrations increased from a concentration of 280 parts per million (ppm) in 1850 to 410 ppm in 2019 (Lee *et al*., 2023) and are predicted to reach between 540 and 970 ppm by 2100 (Stocker & Qin, 2013). Elevated CO_2_ levels alter the nutritional status of plants and therefore could alter immune responses of phytophagous insects (Fig. 2). For example, *Eucalyptus tereticornis* plants grown under elevated CO_2_ (640 ppm) showed reduced foliar nitrogen concentration, increased C:N ratio, and reduced protein and amino acid concentrations compared to plants grown under ambient CO_2_ (400 ppm) (Gherlenda *et al*., 2016). Changes to plant nutritional value can modulate energy availability for immune responses in phytophagous and nectar‐feeding insects (Cotter *et al*., 2011). *Paropsis atomaria* beetles feeding on *E. tereticornis* seedlings grown under elevated CO_2_ (640 ppm) showed increased melanisation but reduced PO activity and haemolymph protein concentrations (Gherlenda *et al*., 2016). The reduction in haemolymph protein concentrations was correlated with reduced foliar N concentrations (Gherlenda *et al*., 2016), suggesting that the change in foliage quality caused by elevated CO_2_ levels contributed to the observed alterations in immune response. Reduced plant nutrition under elevated CO_2_ levels can reduce immunocompetence against parasites and pathogens. *Helicoverpa armigera* larvae raised on wheat grown in elevated CO_2_ levels (750 ppm) and parasitised with *Microplitis mediator* showed a reduced total hemocyte count, impaired hemocyte spreading capacity and reduced encapsulation ratio compared to those reared on wheat grown at ambient CO_2_ (375 ppm) (Yin *et al*., 2014).

Elevated CO_2_ levels can also lower the concentration and diversity of plant secondary metabolites that can function as “exogenous immunity” by protecting insects from pathogens and parasites (de Roode, Lefèvre & Hunter, 2013; Singer, Mason & Smilanich, 2014; Muchoney *et al*., 2022). Monarch butterflies (*Danaus plexippus*) reared on milkweeds grown at a predicted future CO_2_ concentration (760 ppm) showed reduced tolerance – up to 77% – to *Ophryocystis elektroscirrha* parasite infection compared to those reared on milkweed grown at a CO_2_ concentration of 400 ppm (Decker, de Roode & Hunter, 2018). Moreover, elevated CO_2_ increased virulence of the parasites and reduced the hosts' lifespan by approximately 25% (Decker *et al*., 2018). Elevated CO_2_ reduced levels of medicinal cardenolides in milkweed plants and the reduction of lifespan was mainly driven by this lower availability of the medicinal phytochemicals that the butterflies utilise to combat the parasites (Decker *et al*., 2018). Uninfected monarch butterflies showed reduced PO activity and hemocyte and granulocyte concentration (cells/μL) when reared on milkweed grown at elevated CO_2_ (810 ppm) compared to those reared on milkweed grown at 410 ppm (Decker *et al*., 2021). When infected with *O. elektroscirrha* parasites, however, monarchs reared on milkweed grown at elevated CO_2_ showed increased immune responses (PO activity, total PO activity, hemocyte concentration and granulocyte concentration) (Decker *et al*., 2021). This increased immune response may be necessary to compensate for the reduced availability of plant‐derived phytochemicals when grown at elevated CO_2_, however, increased immune expression will have physiological costs or potential costs associated with immunopathology.

### (2) Landscape change

Deforestation, urbanisation, and intensive agriculture lead to habitat loss and landscape change which is one of the major contributors to global insect decline (Winfree *et al*., 2009; Goulson *et al*., 2015). Degraded landscapes support lower abundance and richness of larval and adult food resources both of which contribute to declining populations of pollinators (Carvell *et al*., 2006; Scheper *et al*., 2014; Palash *et al*., 2022). Experimental studies show that dietary restriction in larval (food‐deprived for 5.25 ± 0.04 days) and adult stages (half‐calorie diet) reduced PO activity and hemocyte concentration in the monarch butterfly (McKay, Ezenwa & Altizer, 2016). Additionally, a lack of diversity in the diet can impact infection clearance and survival. For example, parasitised bees fed with a polyfloral blend lived longer than bees fed with monofloral pollen (Pasquale *et al*., 2013). Consequently, lower availability of floral resources in degraded landscapes may lead to a reduced immune response and greater pathogen loads in insects (Pasquale *et al*., 2013; Ricigliano *et al*., 2019; McNeil *et al*., 2020; Fig. 2).

Recent studies have shown that bees in apiaries surrounded by primarily non‐agricultural forage have higher expression of immune genes (i.e. genes for abaecin, apidaecin, defensin 2, lysozyme 2) compared to apiaries surrounded by more intensively cultivated agricultural lands (Ricigliano *et al*., 2019). Similarly, feral bee colonies with access to a greater variety of floral resources and varied habitats expressed immune genes at twice the levels of managed bees in agricultural monocultures following an immune challenge (Youngsteadt *et al*., 2015). Feral bees and apiaries surrounded primarily by non‐agricultural vegetation most likely encounter a greater variety of pathogens which could contribute to their increased expression of immune‐related genes. Pathogen abundance and transmission in combination with reduced insect immunity could therefore be contributing to population declines in degraded landscapes. For instance, pathogen load in bees was greater and survival was lower in highly urbanised environments compared to landscapes with low urbanisation, primarily driven by greater transmission of pathogens rather than differences in bee immunity (Youngsteadt *et al*., 2015). Overall, it is likely that anthropogenic landscape degradation may lead to reduced insect immune defences and thus be contributing to the global pollinator decline (Fig. 2). Landscape change driven by anthropogenic activities is also contributing to dietary shifts or expansions of herbivorous insects. For example, many herbivores have incorporated introduced plants into their diets (Graves & Shapiro, 2003). Because host plant species of herbivorous insects influence their immune response, such dietary changes could impact insect immune defence in unknown ways. The impact of habitat degradation on immunity is not limited to pollinators and nectar‐feeding insects. Because immune responses are condition dependent, it seems likely that habitat degradation could lead to reduced immune responses in insect species where habitat degradation is associated with reduced food availability. Further studies of the impacts of anthropogenic habitat degradation on insect immune defences and how these may contribute to insect population declines are needed.

### (3) Pollution

#### (a) Heavy metals

Anthropogenic activities such as mining, industrial emissions, irrigation and sewage treatments release heavy metal pollutants into the environment (Mohammed, Kapri & Goel, 2011). These heavy metals are non‐biodegradable, accumulate *via* the food chain and therefore can impact immune responses and disease resistance in both terrestrial and aquatic insects (Jiang *et al*., 2021; Singh *et al*., 2022). Mangahas, Murray & McCauley, 2019; Wu *et al*., 2022; Fig. 2). For example, cadmium (Cd) exposure in gypsy moth *Lymantria dispar* reduced total hemocyte numbers, encapsulation and phagocytosis (Wu *et al*., 2022). Furthermore, Cd exposure reduced expression of genes in the IMD, Toll and JAK/STAT pathways, and consequently also reduced expression of AMPs such as cecropin and lebocin (Wu *et al*., 2022). Heavy metal stress has also been shown to affect resistance against pathogens (Dubovskiy *et al*., 2011). For instance, dietary nickel (Ni) reduces survival of *G. mellonella* larvae against the fungus *B. bassiana*; infected larvae fed on a diet containing Ni showed greater mortality compared to infected larvae fed a control diet (Dubovskiy *et al*., 2011).

The impact of heavy metals depends on the dosage and on developmental stage. In *G. mellonella* larvae, a low dose (5 μg/g) of chromium (Cr) and lead (Pb) increased the levels of immune effectors (total hemocyte count, phagocytic activity, extent of encapsulation) and hemolymph immune enzyme activities (acid phosphatase, alkaline phosphatase and PO) whereas the highest doses (100 μg/g) of Cr and Pb inhibited them (Wu & Yi, 2015). Similarly, larvae of the moth *Epirrita autumnata* showed greater encapsulation activity when low doses of Ni and copper (Cu) were present in their diet, however, encapsulation decreased at high dietary levels of Cu and Ni (van Ooik, Pausio & Rantala, 2008). The impact of heavy metal exposure on insect immune responses might also change during development. For example, *Carabus* (*Chaetocarabus*) *lefebvrei* raised in soil polluted with heavy metals showed reduced expression of PO activity in larval and pupal stages but not in the adult stage (Talarico *et al*., 2014).

#### (b) Insecticides

Insecticides are agrochemicals used for controlling pest insects, but often have negative effects on non‐target species. Insecticides impact insect growth (Smith *et al*., 2020; Stuligross & Williams, 2021), behaviour (Smith *et al*., 2020; Siviter & Muth, 2020; Parkinson, Fecher & Gray, 2022), reproductive output (Stuligross & Williams, 2020) as well as immunity (Collison *et al*., 2016; Harwood, Prayugo & Dolezal, 2022) (Fig. 2).

Insecticides such as imidacloprid (a common neonicotinoid targeting the nervous system of insects) dysregulates immune activation pathways. In *Drosophila melanogaster*, imidacloprid dysregulates the IMD pathway which consequently inhibits intestinal dual oxidase (Duox) expression and reduces H_2_O_2_ production (Chmiel *et al*., 2019). Insecticide exposure also affects the ability to induce immune responses: in the stingless bee *Nannotrigona perilampoides* and honey bee *A. mellifera*, exposure to field‐realistic concentrations of the insecticide imidacloprid led to reduced expression of the AMPs abaecin, defensin1, and hymenopteacin (Wu *et al*., 2017; Al Naggar *et al*., 2022a). Exposure to the neonicotinoids thiacloprid, imidacloprid, and clothianidin reduced total hemocyte number, encapsulation response, and antimicrobial activity in *A. mellifera* (Brandt *et al*., 2016). Exposure to insecticides can also impact pathogen susceptibility by impacting host immune responses. The neonicotinoid clothianidin inhibits the nuclear factor‐kappa B (NF‐κB) immune signalling pathway, and as a consequence promoted replication of the viral pathogen deformed wing virus (DWV) in honey bees (Di Prisco *et al*., 2013). Similarly, sublethal doses of flupyradifurone (Al Naggar & Baer, 2019) and imidacloprid (Pettis *et al*., 2012) increased the load of the gut parasites *Nosema* spp. in honeybees. Insecticides also impact interactions between pathogens. Chronic exposure to sublethal doses of imidacloprid resulted in higher infection by DWV in *Nosema ceranae*‐parasitised bees compared to non‐parasitised bees (Balbuena *et al*., 2023).

It is clear that insecticides can impact infection outcome and contribute to greater mortality. In bees parasitised with *Nosema ceranae*, greater mortality was observed when bees were exposed to sublethal doses of imidacloprid for long periods (Balbuena *et al*., 2023) or even during a short exposure to a sublethal dose of flupyradifurone (Al Naggar & Baer, 2019). Reduced survival of infected insects when exposed to insecticides could result from reduced pathogen clearance capacity. For example, honey bee *A. mellifera* can clear infections of the bacteria *Bacillus badius* and *Ochrobactrum anthropic* but coinfection reduced survival following exposure to sublethal dose of thiamethoxam (Decio *et al*., 2021).

Insecticides such as imidacloprid also disrupt the insect gut microbiome composition (Chmiel *et al*., 2019; Fig. 2). Disrupted symbioses could weaken the immune response and promote pathogen susceptibility (Daisley *et al*., 2020a). *D. melanogaster* exposed to imidacloprid displayed greater pathogen susceptibility when infected with the bacterial pathogen *Serratia marcescens* under heat stress (Daisley *et al*., 2017). This greater susceptibility was partly governed by altered symbioses, and could be mitigated by supplementation with *Lactobacillus plantarum* (Daisley *et al*., 2017).

#### (c) Herbicides

Herbicides are widely used in agriculture. In addition to their effects on plants, they can also affect insect development (Defarge, Otto & Hilbeck, 2023), learning (Nouvian, Foster & Weidenmüller, 2023), foraging (Godara *et al*., 2023), gut microbiota (Helander *et al*., 2023) and host–pathogen interactions (Vommaro, Giulianini & Giglio, 2021; Smith *et al*., 2021; Motta, Powell & Moran, 2022; Fig. 2).

Recent studies have provided evidence that herbicides used at the field‐recommended dose can impair insect immunity. For example, exposure of *Harpalus rufipes* beetles to the field‐recommended dose of a pendimethalin‐based herbicide (4 l/ha) led to reduced numbers of circulating haemocytes and phagocytic index (Vommaro *et al*., 2021). Herbicide exposure also reduces the ability to clear pathogens, which could result in greater mortality in infected insects (Fig. 2). Glyphosate is the most widely used commercial herbicide, and exposure to it inhibited PO activity and increased the numbers of the malaria‐causing parasite *Plasmodium falciparum* in *Anopheles gambiae* mosquitoes (Smith *et al*., 2021). Similarly, glyphosate exposure inhibited PO activity, and decreased the size of melanised nodules (which function to eliminate fungal infections) formation in the hemolymph of *G. mellonella* caterpillars (Smith *et al*., 2021). *Cryptococcus neoformans* fungal infection reduced survival of glyphosate‐treated caterpillars compared to saline‐injected and full controls (Smith *et al*., 2021). In another study it was found that glyphosate exposure reduced melanisation and expression of AMPs (apidaecin, defensin and hymenoptaecin) (Motta *et al*., 2022) and decreased the abundance of the symbiotic bacteria *Snodgrassella alvi* (Motta *et al*., 2020) in *Apis melifera*. Consequently, glyphosate‐exposed bees when infected with *S. marcescens* exhibited increased mortality under laboratory conditions but this effect was not found in field experiments (Motta *et al*., 2020).

#### (d) Fungicides

Fungicides are used in agriculture to protect plants from fungal diseases and represent 35% of global pest control chemicals (Zubrod *et al*., 2019). Fungicides are often applied during flowering and as consequence pollinators come into direct contact with them during foraging (Schuhmann *et al*., 2022). Moreover, fungicide applications tend to be frequent and high concentrations of fungicides leach into the aquatic system and soil (Zubrod *et al*., 2019), potentially impacting the health of both aquatic and soil insects. Field‐realistic concentrations of fungicides are considered to be of low toxicity and rarely to cause illness or death of insects. Yet, possible sublethal impacts of fungicides largely have been overlooked. Recent studies have reported sublethal effects of fungicides on insect development (Domingues *et al*., 2021; Xiong *et al*., 2023), foraging (David, Henry & Sprayberry, 2022; DesJardins *et al*., 2024), metabolism (Degrandi‐Hoffman *et al*., 2015; Xiong *et al*., 2023) and immune function (Kaur *et al*., 2023; Xiong *et al*., 2023).

In *A. melifera*, topical exposure of larvae to 50 ppm of the fungicide difenoconazole decreased numbers of granulocytes 1 h after treatment, but this number increased 24 h after treatment (Kaur *et al*., 2023). Similarly, honey bee larvae fed field‐realistic concentrations of pyraclostrobin (100 and 83.3 mg/L) showed impaired immune responses in both larval and pupal stages (Xiong *et al*., 2023). In larvae, pyraclostrobin induces expression of the AMPs defensin1 and hymenoptaecin, but it decreases expression of apidaecin, and abaecin. By contrast, in pupae, increased expression of apidaecin, and hymenoptaecin, but decreased expression of defensin1 was observed (Xiong *et al*., 2023). An impaired immune response was also detected in adult bees fed on chlorothalonil fungicides. Adult bees fed on 5% chlorothalonil solution showed reduced expression of the immune deficiency (*imd*) gene compared to bees feed on standard sucrose water (Conradie *et al*., 2024). Fungicide exposure can also lead to increased infection following exposure to pathogens. For example, honey bee colonies feed on field‐realistic concentrations of boscalid and pyraclostrobin fungicides had higher titres of deformed wing virus and black queen cell virus compared to bees fed on a standard diet (Degrandi‐Hoffman *et al*., 2015). Together, these recent studies provide evidence that fungicides can impact insect immune responses and infection susceptibility; however, most work has focused on honey bees and impacts on other insects remain unknown.

#### (e) Pharmaceuticals

The presence of micropollutants such as pharmaceuticals and personal care products in the environment is an emerging threat, especially for aquatic species (Wang *et al*., 2021). Recent studies have shown that pharmaceuticals can reduce growth, development, reproduction, and immune defence even at very low concentrations in many invertebrates (Gust *et al*., 2013; Ren *et al*., 2022). Investigations on the effects of pharmaceuticals on the insect immune system are currently rather limited, despite their potential to have adverse effects on insect immunity and resistance (Fig. 2).

Pharmaceuticals such as antibiotics are often used in bee colonies to prevent bacterial disease (e.g. foulbrood) of bee larvae (Genersch, 2010). Antibiotics, however, can impact the gut microbiota that has a role in the insect immune system and provides protection from infections (Horak, Leonard & Moran, 2020; Lang *et al*., 2021). *A. melifera* bees fed 450 μg/ml of tetracycline showed a reduced abundance of the gut symbionts *Lactobacillus* Firm‐5, and *Lactobacillus* Firm‐4 *and S. alvi* (Raymann, Shaffer & Moran, 2017). When the bees were challenged with the pathogenic bacterium *Serratia* kz11, tetracycline‐fed bees showed reduced survival compared to bees on a standard antibiotic‐free diet (Raymann *et al*., 2017). Another study in *A. melifera* showed that topical exposure to oxytetracycline depleted the symbionts *Frischella perrera* and *Lactobacillus* Firm‐5 strains in bees and reduced antimicrobial capacity of bee hemolymph against *Arthrobacter globiformis* by 31.27% (Daisley *et al*., 2020b). This study further showed that in‐hive supplementation with three immunostimulatory *Lactobacillus* strains could mitigate antibiotic‐associated microbiota dysbiosis and immune deficits in adult workers (Daisley *et al*., 2020b).

Another widely used pharmaceutical is the painkiller ibuprofen. When administered at concentrations of 0.01, 1, and 100 μg/L to the harlequin fly *Chironomus riparius* larva, ibuprofen led to increased expression of immune genes such as proPO and defensin 96 h after exposure (Muñiz‐González, 2021). As concentrations of ibuprofen between 1 and 100 μg/L have been reported from water bodies across the world (Chopra & Kumar, 2020), it might have a detrimental impact on insect immunity and fitness. Exposure to the veterinary antiparasitic drug ivermectin elevated basal PO activity in yellow dung flies (*Scathophaga stercoraria*) (West & Tracy, 2009). Whether the reported increases in AMPs and basal PO activity induced by exposure to pharmaceuticals affects insect resistance against pathogens has not yet been tested. Activation of the immune response is costly and insects might have to trade this cost against other physiological functions such as growth, development and reproduction (Fig. 2). The immunopathological and physiological costs of pharmaceutical‐induced immune system activation still require investigation.

#### (f) Microplastics

Microplastics are one of the most abundant anthropogenic pollutants. They are released from car tyres, and also from industrial and domestic sources, and are considered a major threat to biodiversity and ecosystem functioning (Lim, 2021; Schell *et al*., 2022). Recent studies have shown that microplastics can have detrimental impact on the immune system of aquatic invertebrates (Détrée & Gallardo‐Escárate, 2018; Gardon *et al*., 2020) as well as terrestrial and aquatic insects. Ingestion of polyethylene microplastic increased basal PO activity in aquatic *C. riparius* larvae (Silva *et al*., 2021a). In the absence of infection, unnecessary activation of immune responses may reduce host fitness. Polyethylene microplastic may damage the gut epithelium which activates wound repair and PO activity as well as increasing ROS levels (González‐Santoyo & Córdoba‐Aguilar, 2012; Silva *et al*., 2021a). Polyethylene microplastic exposure reduces the energy available for other functions in *C. riparius* larvae, which have to allocate resources to the cost of upregulated immune activity and repairing oxidative damage (Silva *et al*., 2021b). Microplastics have also been reported to alter expression of immune genes and AMPs: polystyrene microplastic exposure in silkworm *Bombyx mori* led to increased expression of lysozyme and cecropin (Muhammad *et al*., 2021).

Ingestion of microplastics can also alter host–pathogen interactions. For instance, exposure to polystyrene microplastic promoted susceptibility to Israeli acute paralysis virus infection in *A. melifera* and *A. cerana* honey bees (Deng *et al*., 2021). Infected bees exposed to microplastic had higher viral loads and exhibited greater mortality compared to bees that were not exposed to microplastic (Deng *et al*., 2021). The impact of plastic on resistance to pathogens is complex and may depend on particle size. While exposure to polystyrene microplastic (5–5.9 μm) increased the survival of silkworm larvae infected with *S. marcescens* compared to controls, exposure to polystyrene nanoplastic (50–100 nm) increased mortality (Muhammad *et al*., 2021).

## VI. INSECT IMMUNITY UNDER MULTIPLE STRESSORS

The anthropogenic factors that impact insect immunity are often studied separately, presumably due to logistical reasons, however, these factors often act in combination. It is crucial to understand whether combinations of these factors act antagonistically, mitigating impacts, or synergistically thereby exacerbating negative consequences on insect immune response and pathogen resistance.

Malathion (an organophosphate insecticide) increases expression of the AMP defensin in yellow fever mosquito *A. aegypti* larvae reared at 20 and 25 °C (Muturi, 2013). However, the reverse pattern was seen when larvae were reared at 30 °C, with reduced expression of defensin (Muturi, 2013). The combination of elevated temperature and exposure to insecticides therefore appears to reduce the energy available to invest in immune defence. This was also the case in *I. elegans* damselflies, where exposure to the insecticide chlorpyrifos reduced energy availability under conditions of high mean temperature combined with high daily temperature fluctuations (Verheyen & Stoks, 2020).

Studies that combine multiple stressors often provide a more realistic picture of the impacts of global change (Rillig *et al*., 2019). In one study, investment in food intake, and immune response were measured under experimental heatwaves in *I. elegans* damselfly larvae (Van Dievel, Stoks & Janssens, 2017). Heatwave‐exposed damselfly larvae increased food intake and exhibited greater PO activity and melanisation (Van Dievel *et al*., 2017). In *C. puella* damselflies the impact of heatwaves was studied in combination with food limitation and pesticide (chlorpyrifos) exposure (Dinh, Janssens & Stoks, 2016). Neither food limitation nor exposure to the pesticide chlorpyrifos individually reduced PO activity (Dinh *et al*., 2016). However, in heatwave‐exposed damselflies, both pesticide exposure and food limitation led to decreased PO activity (Dinh *et al*., 2016). Similarly, glyphosate at field‐realistic doses reduced the prevalence of the malaria parasite in *Culex pipiens* mosquitoes raised on a standard diet (Bataillard, Christe & Pigeault, 2020). However, under nutritionally stressed conditions, glyphosate in the diet had the opposite effect, increasing the prevalence of the malaria parasite in mosquitoes (Bataillard *et al*., 2020). These studies provide evidence for complex interactions between anthropogenic stressors that require detailed future investigation.

## VII. INSECT IMMUNITY AND EXTINCTION RISK IN THE ANTHROPOCENE

The previous sections discussed how anthropogenic factors such as anthropogenic climate change, landscape alterations, and pollutants can impact insect immune responses, and can therefore alter host–pathogen interactions. Some factors such as increasing temperature, heatwaves, landscape change, and pollutants seem to inhibit the ability of insects to induce an immune response upon pathogen exposure and thereby will reduce insect fitness by promoting pathogen‐driven mortality (Fig. 2). On the other hand, some anthropogenic factors such as microplastics may mimic pathogen particles and activate insect immune responses (Fig. 2). Such non‐specific immune activation will deplete energy reserves, with a corresponding reduction in energy available for other fitness traits such as reproduction. Anthropogenic factors therefore likely increase pathogen‐driven mortality and reduce investment in other fitness traits and thus reduce overall fitness (Fig. 2). It is noteworthy that some important effects may be more long term, and thus may not be detected by standard toxicological testing. A study in bumble bees showed that while low concentrations of a pesticide had no impact on worker survival, the production and survival of new queens in the next year was severely curtailed (Whitehorn *et al*., 2012).

Fitness costs imposed by altered host–pathogen interactions under global change can contribute to local population collapse (LeBrun *et al*., 2022) and global insect decline (Fig. 2). Recent studies have provided strong evidence that anthropogenic activities are a major contributor to species extinction (Goulson *et al*., 2015; Wagner, 2020; Wagner *et al*., 2021; St. Leger, 2021; Harvey *et al*., 2023). Insect populations are globally declining in response to lethal effects of heatwaves, extreme temperatures, flood, drought, wildfires and habitat loss. Global change drivers also can impair insect immune defences and thereby increase direct pathogen‐driven mortality. In addition, the distribution range of pathogens could expand under changing climates and insects therefore could encounter new pathogens. Increasing temperatures almost certainly promote the growth and development of pathogens and consequently their virulence in a species‐specific fashion (Shapiro & Cowen, 2012; Bruneaux *et al*., 2022). To protect themselves from novel pathogens, insects may have to invest more in their immune responses, with a consequent reduction in investment into other fitness traits such as reproduction. Such reductions of insect fitness are often overlooked when determining the impacts of anthropogenic stressors. The overall fitness costs that stem from overinvestment in immunity may have synergistic effects on other extinction‐causing elements and have the potential to exacerbate current extinction trends.

## VIII. INSECT IMMUNITY AND VECTOR‐BORNE DISEASES IN THE ANTHROPOCENE

Insect‐transmitted vector‐borne diseases are increasing in prevalence because of anthropogenic global change (Caminade, McIntyre & Jones, 2019; Franklinos *et al*., 2019; Nova *et al*., 2022; Fig. 2). The abundance of some vectors is increasing as a result of range expansion and greater reproductive success aided by artificial breeding microhabitats such as plastic containers (Banerjee, Aditya & Saha, 2015; Ryan *et al*., 2019; Franklinos *et al*., 2019). Climate change, plastic pollution, pesticides and land cover change thus may be contributing to the rise of insect‐transmitted diseases by increasing vector abundance but also by impacting insect–pathogen interactions (Franklinos *et al*., 2019; Bellone & Failloux, 2020; Rocklöv & Dubrow, 2020; Loiseau & Sorci, 2022; Fig. 2). West Nile Virus epidemics have been found to be associated with drought which was correlated with mosquito infection prevalence rather than mosquito abundance (Paull *et al*., 2017). Similarly, global warming is associated with higher malaria prevalence; in highland Columbia and Ethiopia higher malaria prevalence was observed in warmer years (Siraj *et al*., 2014). In warmer temperatures insects have lower pathogen clearance ability, resulting in increased prevalence of infected insects in a population and thereby increased chances of disease transmission. When field‐caught *Aedes japonicus* were orally infected with Zika virus and incubated at 21, 24, or 27 °C, the mosquitoes incubated at 27 °C had a greater infection rate (66.7%) and higher viral titre (log_10_ RNA copies 5.9/specimen) than those incubated at 21 °C (infection rate 10% and viral titre log_10_ RNA copies 4.6/specimen) (Jansen *et al*., 2018; Fig. 3). *C. pipiens* incubated at a higher temperature (30 °C) had higher infection rates compared with mosquitoes incubated at a lower temperature (18, 20 and 26 °C), even though they were given the same blood meal infected with West Nile virus (Dohm, O'Guinn & Turell, 2002; Fig. 3). In addition to infection rates, temperature can also impact virus transmission; in *Aedes albopictus* mosquito Dengue virus 2 transmission from the midgut to the salivary gland was more rapid when mosquitoes were incubated at a higher temperature (32 °C) than at 23 and 28 °C, and transmission was not detected in mosquitoes that had been incubated at 18 °C (Liu *et al*., 2017).

Other anthropogenic factors such as urbanisation, insecticides and microplastics impact vector immunity and therefore could affect the prevalence of vector‐transmitted diseases (Misslin *et al*., 2016; Loiseau & Sorci, 2022). Urbanisation impacts insect immunity (Youngsteadt *et al*., 2015; Tüzün & Stoks, 2021). Recent studies have provided strong associations between urbanisation and the prevalence of mosquito‐borne diseases such as dengue which is primarily driven by habitat suitability for mosquitoes but could also be affected by the reduced immunity of vectors in urbanised areas (Misslin *et al*., 2016). Microplastics are predicted to accelerate the spread of infectious diseases partly *via* promoting the growth of pathogens but also by impacts on mosquito microbiota (Fig. 2), which can provide defence against malaria parasites (Dong, Manfredini & Dimopoulos, 2009). Ingestion of microplastics has been shown to decrease species diversity of bacterial and fungal microbiota in *A. aegypti* and *A. albopictus* mosquitoes (Edwards *et al*., 2023). The impact of anthropogenic factors on vector‐transmitted diseases remains largely unknown due to the lack of studies directly investigating how anthropogenic factors impact insect immunity and pathogen or parasite loads in vectors and their transmission capacities. While there is evidence that anthropogenic factors impact vector immunity, data on how these modifications impact vector–pathogen interactions is scant and more studies are required.

## IX. FUTURE DIRECTIONS

**Fig. 4:**
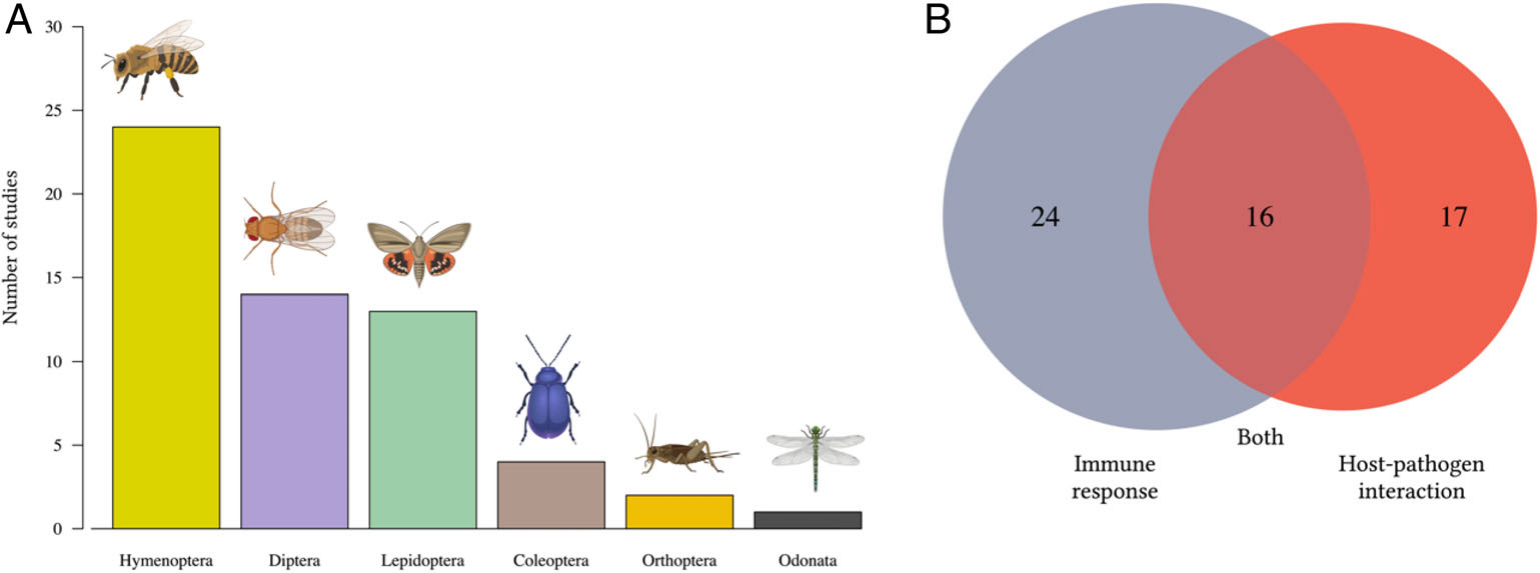
(A) Overview of the taxonomic distribution of studies on insect immunity included in the review. (B) Venn diagram showing number of studies that measured immune response, pathogen resistance, or both. Figure created in R version 4.2.1 (R Core Team, 2022) using R packages VennDiagram (Chen, 2022) and Durga (Khan & McLean, 2024).

The impact of anthropogenic changes on insect immunity varies across landscapes and among taxa. Species from different ecosystems are adapted to their local pathogens (Schmid‐Hempel, 2005; Grüter, Jongepier & Foitzik, 2018; Ferguson & Adamo, 2023). Global change will disrupt this balance and the extent of this disruption might vary among species. It will be important to determine how the immunity of different species is impacted by anthropogenic changes to identify those species that are most vulnerable to global change. Most studies to date on the impacts of anthropogenic activities on insect immunity have focused on model systems (e.g. fruit flies, honey bees, mosquitoes) and on species from the orders Hymenoptera, Diptera and Lepidoptera (Fig. 4A). Multispecies comparative studies of immune responses and pathogen resistance of insects in response to multiple anthropogenic stressors will be useful to determine the extinction risks posed by pathogens.

The impact of anthropogenic factors on insect immunity is complex, and ideally requires the investigation of all aspects: immune responses, pathogen resistance and fitness. Studies investigating anthropogenic impacts on insect immunity often assess either immune responses or pathogen resistance, but not both (Fig. 4B), with only 26.31% of studies in our data set measuring both immune responses and pathogen resistance (Fig. 4B). Studying only immune responses or pathogen resistance will provide an incomplete picture. For example, while microplastic exposure is known to increase expression of immune genes (Silva *et al*., 2021a; Muhammad *et al*., 2021; Deng *et al*., 2021), it is not known how it impacts pathogen resistance, energetic expenditure or immunopathology.

Recent studies have provided evidence that gut symbionts stimulate the insect immune system and provide protection from infection (Horak *et al*., 2020; Steele *et al*., 2021). Pesticides, urbanisation, pollution and heatwaves all have detrimental impacts on symbionts and can break down insect–gut microbe symbiosis (Motta, Raymann & Moran, 2018; Motta *et al*., 2022; Villabona *et al*., 2023; Fig. 2). However, currently evidence linking these two phenomena is scarce.

How insect immune systems will evolve in the Anthropocene remains unclear (Urban *et al*., 2024). Whether phenotypic plasticity or microevolution of insect immune systems will be able to mitigate negative impacts of anthropogenic factors on insect immunity is unknown. An experimental evolution approach, in which insects are exposed to anthropogenic factors and any effects of selection on immunity and resistance are measured, would provide a clearer picture of how microevolution could impact insect immunity.

The extent of anthropogenic changes differs across climatic zones and habitats, and understanding how this impacts disease‐driven extinction risk for insects across landscapes will be essential. Obtaining evidence of how insect immunity varies across landscapes will be important to understand how vector‐borne diseases in humans might vary in prevalence in the future.

Anthropogenic factors impact the ability of insects to mount an immune response but also the ability of pathogens to circumvent insect defences and to survive in the environment (Linder *et al*., 2008; Apidianakis & Rahme, 2009; St. Leger, 2021). It will be important therefore to determine the impact of anthropogenic factors on both host immune response and pathogen virulence to understand how anthropogenic factors impact host–pathogen interactions.

Components of global change such as increasing temperatures, insecticides and pollutants are often studied separately rather than in combination, although insects are often simultaneously exposed to a combination of several stressors. Future studies should focus on the impacts of combined stressors on insect immunity.

A set of open research questions to improve our understanding of the impact of anthropogenic global changes on insect immunity are provided in Table 2.

**Table 2:** Open research questions for studies on the impact of anthropogenic global changes on insect immunity.

1	How does global change impact cellular and molecular mechanisms of insect immune responses?
2	How does the impact of anthropogenic factors on insect immune responses vary across species, taxa and between hemi‐ and holometabolous insects?
3	How does climate change impact host–pathogen interactions across habitats and landscapes?
4	How do anthropogenic factors impact pathogen virulence?
5	To what extent do pathogen virulence and host immune responses contribute to the altered disease landscape in the Anthropocene?
6	Will anthropogenic factors affect host tolerance towards pathogens?
7	How phenotypically plastic is the insect immune response and to what extent can plasticity mitigate the impact of anthropogenic factors on insect immune responses and insect–pathogen interactions?
8	Do multiple anthropogenic factors act antagonistically or synergistically on insect immune responses and host–pathogen interactions?
9	What are the immunopathological and fitness costs of immune‐activating anthropogenic stressors such as microplastics and some pharmaceuticals? Can immune activation by anthropogenic stressors provide protection against pathogens?
10	How do global change factors impact host–symbiont interactions and microbiome‐mediated immune protection?

Overall, accumulating evidence shows that anthropogenic factors have a detrimental impact on insect immune defences and hence promote transmission of vector‐borne diseases. It is therefore urgent to apply initiatives to mitigate this impact. Mitigating some anthropogenic factors such as climate change will require long‐term strategies, but some aspects, such as insecticide use or pollution by microplastics and pharmaceuticals can be mitigated more rapidly. For example, research initiatives to replace insecticides with environmentally friendly alternatives should be promoted. Plastics could be replaced with biodegradable materials, although these “environmentally friendly” alternative materials also need to be tested for any impacts on insect immunity and fitness. These approaches could be implemented at a regional or national scale and could be targeted to protect locally vulnerable species or restrict the spread of vector‐borne diseases across endemic regions.

## X. CONCLUSIONS

(1) Anthropogenic factors, such as insecticides and heavy metals, directly impact insect immune responses by inhibiting immune activation pathways.
(2) Anthropogenic factors such as global warming or heatwaves increase insect energy demands and thus result in reduced energy availability for immune responses.
(3) Elevated CO_2_ and landscape degradation reduce food quantity/quality thereby reducing resources for mounting immune responses.
(4) Factors such as pharmaceuticals and antibiotics activate insect immune responses, independent of an infection.
(5) Anthropogenic factor‐driven suppressed immune responses reduce fitness by causing disease‐driven mortality in the presence of infection.
(6) Anthropogenic factor‐driven elevated immune responses reduce energy available to invest in other fitness traits such as reproduction.
(7) Anthropogenic factors may act synergistically or antagonistically. The combined impact of multiple stressors on immune response and pathogen resistance has rarely been studied but would almost certainly provide a broader understanding of insect immunity in the Anthropocene.
(8) Until now, impacts of anthropogenic activities on insect immunity have mostly been studied on model systems (e.g. fruit flies, honey bees, mosquitoes) and impacts of anthropogenic factors on species other than the orders Hymenoptera, Diptera and Lepidoptera remain largely unexplored.
(9) Impacts of anthropogenic factors on insect immunity may promote vector‐borne diseases.
(10) Anthropogenic factors can contribute to species extinction *via* pathogen‐driven mortality and reduced reproductive output.
